# Supplementary material for: Genetic analysis of body weight in wild populations of medaka fish from different latitudes
Source: PLoS One. 2020 Jun 16;15(6):e0234803. doi: 10.1371/journal.pone.0234803 (PMC7297337; doi:10.1371/journal.pone.0234803)
Supplement: Attachment — Submitted filename: Response to Reviewers.docx [file pone.0234803.s004.docx]

**Responses to the Academic Editor**

*1. Please ensure that your manuscript meets PLOS ONE's style requirements, including those for file naming. The PLOS ONE style templates can be found at http://www.plosone.org/attachments/PLOSOne_formatting_sample_main_body.pdf and http://www.plosone.org/attachments/PLOSOne_formatting_sample_title_authors_affiliations.pdf*

**Response:** We have revised the manuscript and the other files according to the guidelines of PLOS ONE as suggested.

*2. We note that you are reporting an analysis of a microarray, next-generation sequencing, or deep sequencing data set. PLOS requires that authors comply with field-specific standards for preparation, recording, and deposition of data in repositories appropriate to their field. Please upload these data to a stable, public repository (such as ArrayExpress, Gene Expression Omnibus (GEO), DNA Data Bank of Japan (DDBJ), NCBI GenBank, NCBI Sequence Read Archive, or EMBL Nucleotide Sequence Database (ENA)). In your revised cover letter, please provide the relevant accession numbers that may be used to access these data. For a full list of recommended repositories, see http://journals.plos.org/plosone/s/data-availability#loc-omics or* [*http://journals.plos.org/plosone/s/data-availability#loc-sequencing*](http://journals.plos.org/plosone/s/data-availability#loc-sequencing)*.*

**Response:** We have deposited the raw sequence data obtained by RAD sequencing into the DDBJ Sequence Read Archive (DRA) accession numbers DRR226810-DRR226964 (lines 143–145).

*3. Thank you for stating in your Funding Statement:*

*"This work was supported in part by a JSPS KAKENHI “Grant-in-Aid for Scientific Research (C)” (15K07163, 19K06785) to AS and “Grant-in-Aid for Specially Promoted Research” (26000013) and “Grant-in-Aid for Scientific Research (S)” (19H05643) and by the Human Frontier Science Program (RGP0030/2015) to TY. The funders had no role in study design, data collection and analysis, decision to publish, or preparation of the manuscript."*

*Please provide an amended statement that declares *all* the funding or sources of support (whether external or internal to your organization) received during this study, as detailed online in our guide for authors at http://journals.plos.org/plosone/s/submit-now. Please also include the statement “There was no additional external funding received for this study.” in your updated Funding Statement.*

*Please include your amended Funding Statement within your cover letter. We will change the online submission form on your behalf.*

**Response:** We have changed the original statement to: “This work was supported by a JSPS KAKENHI Grant-in-Aid for Scientific Research (C) (15K07163, 19K06785) to AS, Grants-in-Aid for Specially Promoted Research (26000013), and for Scientific Research (S) (19H05643) to TY, and the Human Frontier Science Program (RGP0030/2015) to TY. There was no additional external funding received for this study. The funders had no role in the study design, data collection and analysis, decision to publish, or preparation of the manuscript.”

**Response to Reviewer 1**

*The article is well-written, and the topic quite interesting with potential implications for aquaculture breeding programs. No major modifications are required prior to publication. The only minor modification suggested is related to the introduction. In fact, while the authors recognized that their results could have implications for aquaculture, it seems not strictly related to their research the reference to human diseases and medicine science in the two paragraphs (lines 53-64). This part could be shortened or even omitted.*

**Response:** We have shortened the two paragraphs as suggested (lines 50–57).

**Responses to Reviewer 2**

*Is the manuscript presented in an intelligible fashion and written in standard English?*

*No*

**Response:** We used the service of an English proofreading company to improve clarity.

*I am unfortunately confused with the way the authors approached this subject.
The content of the paper does not help much the readers to understand how the laboratory experiments and the downstream analyses were performed.*

**Response:** We have added more details to facilitate a better understanding of the experimental procedures and downstream analyses.

*1.- in the abstract, the authors have at least a paragraph for obesity research. How is this relevant to the manuscript? Using medaka as a model for human health? We already know that most of these traits are polygenic. Therefore, the results of your study pointing on a single gene for growth might need more thorough reanalysis.*

**Response:** We have deleted the word “medical” from the abstract and have diminished the claim in the Introduction section. We have shortened and revised the two paragraphs that described obesity research (lines 50–57). Furthermore, we have added a description of the relevance and relationships among body weight, growth, and animal health, which affect aquaculture economics.

*2.- For the RAD approach: did you finally use both or a single enzyme? The number of SNPs you finally used (371) is much fewer than that people usually report for RAD or ddRAD approaches. You do not comment on this.*

**Response:** We used both enzymes; we have added the relevant information in the Materials and Methods section (line 124).

We initially obtained 5,019 RAD-sequencing markers that were genotyped in > 70% of all 155 individuals from the parental, F_1_, and F_2_ generations.

In this experiment, F_2_ individuals originated from five parental Maizuru and Ginoza pairs. To obtain informative and reliable markers for QTL analysis, we selected markers with genotyping datasets in the parental, F_1_, and F_2_ generations with homozygous but different genotypes between all Maizuru and all Ginoza parents and a heterozygous genotype in F_1_. We also selected markers that were genotyped in > 80% of the F_2_ fish. After these selection processes, we reduced the number of markers to 371 for QTL analysis.

*3. - Mapping and QTL identification: with the above mentioned number of SNPs it is normal to have a very sparse linkage map with an average of only 15 markers per linkage group or chromosome. To my opinion, this number is very small to have a sound analysis.*

**Response:** Indeed, more markers would increase the statistical power to detect more QTL and be useful for high-resolution mapping. However, we decided to use only reliable markers to minimize or avoid errors.

*4. - On chromosome 4, there are only 11 markers mapped and the one showing a positive association with growth is located at the extreme; from the literature we know that sometimes it might be erroneous.*

**Response:** We designed and genotyped the new marker “ch4_0.68M,” which is located on the distal region of the medaka chromosome 4. The genetic distance between ch4_0.68M and the RAD 56900 marker supported the linkage of these two loci. Therefore, the position of 56900 and the QTL on chromosome 4 is reliable. Genotyping data on the new marker can be found in the supplemental S3 Table and in the revised manuscript (lines 209–211).

*5. - The 12 genes span a region, if I am not mistaken, of approximately 4Mb and the authors have invested a lot of effort and expenses to go for sequencing all these genes for the “source” populations (how many fish exactly?) and identify fixed differences. Very little is reported for the way you performed it; the primers, how many PCR reactions and different fragments per gene etc.*

**Response:** We sequenced the whole genomes of four Maizuru and four Ginoza individuals using HiSeq 4000 (Illumina). Since these whole genome data will be published elsewhere (in preparation), the data cannot be submitted to a public database at this time. However, we showed all the SNP in the coding regions of the 12 genes in all Maizuru and Ginoza individuals included in the variant analyses in the supplemental S2 Table, lines 158–160. After reanalyzing the SNP of these Maizuru and Ginoza individuals, we added more data to Table 2.
